# Symptom Resolution and Meaningful Improvement in Quality of Life With Risankizumab in Patients With Ulcerative Colitis: *Post Hoc* Analysis of the Randomized INSPIRE and COMMAND Studies

**DOI:** 10.14309/ajg.0000000000003420

**Published:** 2025-03-17

**Authors:** Joana Torres, Julian Panés, Corey A. Siegel, Marla C. Dubinsky, Parambir S. Dulai, Satoshi Tanida, Silvio Danese, Jasmina Kalabic, Jyun-Heng Lai, Nidhi Shukla, Leah Rizzo, Cecile Holweg, Dolly Sharma, Remo Panaccione

**Affiliations:** 1 Gastroenterology Division, Hospital Beatriz Ângelo, Loures, Portugal;; 2 Gastroenterology Division, Hospital da Luz, Lisbon, Portugal;; 3 Faculdade de Medicina, Universidade de Lisboa, Lisbon, Portugal;; 4 Hospital Clínic Barcelona, IDIBAPS, CIBERehd, Barcelona, Spain;; 5 Section of Gastroenterology and Hepatology, Center for Digestive Health, Dartmouth Health, Lebanon, New Hampshire, USA;; 6 Dr Henry D. Janowitz Division of Gastroenterology, Icahn School of Medicine at Mount Sinai, New York, New York, USA;; 7 Department of Medicine, Division of Gastroenterology and Hepatology, Northwestern University, Chicago, Illinois, USA;; 8 Education and Research Center for Community Medicine, Nagoya City University Graduate School of Medical Sciences, Nagoya, Japan;; 9 Gastroenterology and Endoscopy, IRCCS Ospedale San Raffaele and Vita-Salute San Raffaele University, Milan, Italy;; 10 AbbVie Deutschland GmbH & Co. KG, Ludwigshafen, Germany;; 11 AbbVie, North Chicago, Illinois, USA;; 12 Health Outcomes Division, College of Pharmacy, The University of Texas at Austin, Austin, Texas, USA;; 13 Inflammatory Bowel Disease Unit, University of Calgary, Calgary, Alberta, Canada.

**Keywords:** risankizumab, ulcerative colitis, patient-reported outcomes, patient-reported symptoms, quality of life

## Abstract

**INTRODUCTION:**

Patients with ulcerative colitis (UC) experience disruptive symptoms that can impair quality of life (QoL). This study examined the effect of risankizumab (RZB) induction and maintenance treatment on symptom resolution and health-related QoL (HRQoL) outcomes.

**METHODS:**

For the 12-week induction, patients were randomized to intravenous (IV) RZB 1,200 mg (RZB1200) or placebo (PBO). For the 52-week maintenance, clinical responders to induction were rerandomized to subcutaneous (SC) RZB 180 mg (RZB180), RZB 360 mg (RZB360), or PBO (RZB withdrawal). This *post hoc* analysis assessed individual and comprehensive resolution of UC-related symptoms and clinically meaningful within-person changes (MWPCs) for HRQoL outcomes.

**RESULTS:**

RZB improved symptomatic outcomes compared with PBO at week 4 (nominal *P* ≤ 0.001; abdominal pain, bowel urgency, fecal incontinence [nominal *P* ≤ 0.01]) through the 12-week induction (*P* ≤ 0.001; abdominal pain [*P* ≤ 0.01]; fecal incontinence, sleep interruption [nominal *P* ≤ 0.001]). Greater improvements were achieved with RZB compared with PBO (RZB withdrawal) for symptomatic outcomes through the 52-week maintenance. More patients treated with RZB achieved comprehensive symptom resolution (complete resolution of all 6 UC-related symptoms) compared with PBO at week 12 of induction (RZB1200, 21.8%; PBO, 9.5% [nominal *P* ≤ 0.001]) and week 52 of maintenance (RZB180, 23.5%; RZB360, 19.4%; PBO [RZB withdrawal], 14.2%; nominal *P* ≤ 0.05 and *P* = 0.1358, respectively). RZB compared with PBO improved MWPCs across HRQoL outcomes at week 12 of induction (nominal *P* ≤ 0.001; work time missed [nominal *P* = 0.0033]). At week 52 of maintenance, RZB180 compared with PBO (RZB withdrawal) improved MWPCs across HRQoL outcomes (nominal *P* ≤ 0.001; overall work impairment [nominal *P* ≤ 0.01], work time missed [nominal *P* = 0.3324], impairment while working [nominal *P* ≤ 0.05]) and more patients treated with RZB360 achieved MWPCs for the Ulcerative Colitis Symptom Questionnaire, Inflammatory Bowel Disease Questionnaire, and the 36-Item Short-Form Survey Physical Component Summary (all nominal *P* ≤ 0.05).

**DISCUSSION:**

RZB treatment improved UC-related symptoms and HRQoL outcomes compared with PBO in patients with UC.

## INTRODUCTION

While clinical activity indexes in ulcerative colitis (UC) clinical trials primarily focus on rectal bleeding and stool frequency, patients with UC often experience other disruptive symptoms such as abdominal pain, bowel urgency, tenesmus, fecal incontinence, nocturnal bowel movements, and sleep interruption (1–5). Symptoms such as abdominal pain and bowel urgency can impair the quality of life (QoL) for patients with UC (1–3). Evidence suggests that both abdominal pain and bowel urgency can contribute to sleep disturbances and daytime fatigue in patients with inflammatory bowel disease (6). Furthermore, poor sleep quality is associated with lower QoL and greater disability (5). Additional symptoms such as tenesmus, fecal incontinence, nocturnal bowel movements, and sleep interruption are important to patients and are rarely captured. These symptoms are not often explored and can contribute to the overall disease burden (7) and, therefore, are vital to address.

Short-term treatment goals for UC per the Selecting Therapeutic Targets in Inflammatory Bowel Disease (STRIDE)-II consensus are to control inflammation and reduce symptoms, whereas long-term goals include endoscopic healing, absence of disability, and normalization of QoL (8). Furthermore, assessing patient-reported outcomes (PROs) provides insight into patients' perspectives on the disease and treatment, which is necessary for the effective management of UC.

Risankizumab (RZB), a humanized immunoglobulin G1 monoclonal antibody that targets the p19 subunit of interleukin-23, has been approved for the treatment of UC (9–12). RZB has demonstrated improved clinical remission rates during induction and maintenance and an acceptable safety profile with RZB vs PBO for the treatment of patients with moderately to severely active UC (9). Following these results, it is important to study the impact of RZB on additional symptoms of patients, such as PROs of abdominal pain and bowel urgency, and other novel outcome measures (i.e., tenesmus, fecal incontinence, nocturnal bowel movements, and sleep interruption).

The aim of this study was to examine the effect of induction and maintenance treatment with RZB compared with placebo on individual UC-related symptoms and comprehensive resolution of all 6 symptoms, as well as clinically meaningful within-person changes (MWPCs) of health-related quality-of-life (HRQoL) outcomes. This study contributes to the existing knowledge by exploring additional novel outcome measures that have not been extensively studied in previous UC trials.

## METHODS

### Study design and population

We analyzed data from 2 phase 3, multicenter, randomized, double-blind, PBO-controlled clinical studies evaluating the efficacy and safety of RZB induction (INSPIRE, NCT03398148) and maintenance (COMMAND, NCT03398135) therapy for adults with moderately to severely active UC. The full trial details, including methods, were previously reported (9). In brief, in the 12-week INSPIRE induction study, patients were randomized 2:1 to receive intravenous (IV) RZB 1,200 mg or PBO. Clinical responders to 12 weeks of RZB IV were randomized 1:1:1 to receive subcutaneous (SC) RZB 180 mg, RZB 360 mg, or PBO (RZB withdrawal) in the 52-week COMMAND maintenance study and were included in the current efficacy analysis.

Full inclusion and exclusion criteria have been published previously (9). Patients aged 18 years or older and 80 years or younger with a diagnosis of UC for ≥3 months, an Adapted Mayo score of 5–9 points, and an endoscopic subscore of 2–3 (confirmed by central review) were included. Patients with a history of intolerance or inadequate response to conventional therapy alone and/or to ≥1 advanced therapy (AT) were included; there was no maximum to the number of ATs the patients could have had exposure to. Patients were excluded if they had prior exposure to p40 inhibitors (i.e., ustekinumab) or p19 inhibitors (i.e., risankizumab, mirikizumab). This analysis also included a subgroup of patients who were categorized by their prior exposure to ATs (e.g., infliximab, adalimumab, golimumab, vedolizumab, tofacitinib, filgotinib, upadacitinib, and ozanimod), including patients with or without an inadequate response (IR) or intolerance (AT-IR or non-AT-IR).

### Outcomes

Information for the individual symptoms was collected daily by an electronic diary. The psychometric validation analyses demonstrating that the abdominal pain and bowel urgency diary items are construct valid, capable of distinguishing between groups known to be clinically different, and sensitive to change over time are described have been previously published (13). Tenesmus, fecal incontinence, nocturnal bowel movements, and sleep interruption are novel diary outcome measures internally developed and validated by AbbVie in accordance with US Food and Drug Administration guidance (14,15) on the use of a PRO instrument to assess an end point in a clinical trial.

The abdominal pain diary item was a single-item questionnaire using a 4-point scale (0 = none, 1 = mild, 2 = moderate, 3 = severe) to evaluate symptom frequency and severity over time. The mean of the scores from the most recent 3 days, and up to 10 days, before each study visit was calculated. Bowel urgency, nocturnal bowel movements, and tenesmus were scored independently with binary response options (no = 0, yes = 1), in which “no” meant the respondent did not experience the symptom within the past 24 hours and “yes” meant the respondent did experience the symptom within the past 24 hours. Scores were determined by calculating the mean of the numeric values over the 3 days, and up to 10 days, before each study visit. Sleep interruption scoring quantified the number of nights with sleep interruption because of UC symptoms in the most recent week (i.e., 7 days) before each study visit. If patients answered “yes” and “due to UC symptoms,” they received a score of 1 for that day; otherwise, patients received a score of 0. Scores were determined by calculating the mean of the numeric values (0 for no sleep interruption due to UC symptoms, and 1 for sleep interruption due to UC symptoms) over the most recent week (i.e., 7 days) and then multiplying the result by 7; therefore, scores ranged between 0 and 7 for each study visit. Fecal incontinence scoring quantified the number of weekly episodes of accidental bowel leakage (e.g., accidental soiling of underwear) before each study visit. Scores were determined by calculating the mean of the number of fecal incontinence episodes over the most recent 7 days before a study visit and then multiplying by 7. The resolution (absence as defined above) of each individual symptom (i.e., abdominal pain, bowel urgency, tenesmus, fecal incontinence, nocturnal bowel movements, or sleep interruption) was assessed, as well as the comprehensive symptom resolution (defined as complete resolution of all 6 UC-related symptoms) and a sensitivity analysis among patients who presented with these symptoms at baseline.

MWPCs for HRQoL outcomes (Functional Assessment of Chronic Illness Therapy-Fatigue [FACITF; MWPC threshold of ≥5-point increase] (16), Ulcerative Colitis Symptom Questionnaire [UCSQ; MWPC threshold of ≥10-point increase] (17), Inflammatory Bowel Disease Questionnaire [IBDQ; MWPC threshold of ≥16-point increase] (18), Work Productivity and Activity Index [WPAI; overall work impairment, MWPC threshold of ≥7.3-point decrease; work time missed, MWPC threshold of ≥6.5-point decrease; impairment while working, MWPC threshold of ≥6.1-point decrease; activity impairment, MWPC threshold of ≥8.5-point decrease] (19), the 36-Item Short-Form Survey Physical Component Summary [SF-36 PCS; MWPC threshold of ≥4.1-point increase] and Mental Component Summary [MWPC threshold of ≥4.1-point increase] (20), and the EuroQoL 5 Dimensions 5 Levels [EQ-5D-5L] questionnaire visual analog scale [VAS; MWPC threshold of ≥10.9-point increase] and index [MWPC threshold of ≥0.076-point increase] (21) were reported. Information for each HRQoL outcome was collected at specified site visits. Safety was not assessed in this analysis but has been published previously (9).

In this *post hoc* analysis, the achievement of individual symptom resolution and MWPC of HRQoL outcomes at week 12 of induction and at week 52 of maintenance were also assessed among patients with prior inadequate response or intolerance to advanced therapy (AT-IR).

### Statistical analyses

The results were reported as adjusted percentage differences of resolution for each individual symptom and for comprehensive symptom resolution, as well as MWPC for each HRQoL outcome for RZB vs PBO and analyzed based on corresponding 95% confidence intervals and *P* values. *P* values at week 12 of induction and week 52 of maintenance for the achievements of no abdominal pain, no bowel urgency, no tenesmus, no fecal incontinence, no nocturnal bowel movement, and no sleep interruption are considered ranked secondary end points and are not *post hoc* analyses. Adjusted differences were calculated based on Mantel-Haenszel common rate difference. Treatment groups were compared using the Cochran-Mantel-Haenszel test adjusted for the following strata: AT-IR status (yes vs no), baseline steroid use (yes vs no), and baseline Adapted Mayo score (≤7 vs >7). The calculations were based on nonresponder imputation incorporating multiple imputation to handle missing data because of logistic restrictions (COVID-19 or geopolitical restrictions) or nonresponder imputation only if there were no missing data because of logistic restrictions. For continuous end points, a mixed-effect model repeated measurement with return-to-baseline multiple imputation was used while adjusting for categorical fixed effects of treatment, visit, and treatment-by-visit interaction; randomization stratification factors (number of prior failed advanced therapies [0, 1, vs >1], baseline steroid use [yes vs no], and baseline Adapted Mayo score [≤7 vs >7]); and the continuous fixed covariates of baseline measurements. MWPCs were measured from induction baseline to induction week 12 and to maintenance week 52 among clinical responders to the 12-week induction.

### Ethics

The Independent Ethics Committee or Institutional Review Board at each study site approved the study protocol, informed consent forms, and recruitment materials before patient enrollment. The studies were conducted in accordance with the International Conference for Harmonization guidelines, applicable regulations, and the Declaration of Helsinki. All patients provided written informed consent before screening.

## RESULTS

### Baseline characteristics

A total of 975 patients (PBO, N = 325; RZB 1,200 mg IV, N = 650) were enrolled in the induction study and 548 (PBO [RZB withdrawal] SC, N = 183; RZB 180 mg SC, N = 179; RZB 360 mg SC, N = 186) in the maintenance study. Baseline disease characteristics and patient demographics were balanced across treatment groups and were previously reported (9). Across treatment groups at induction baseline, 38.2%–40.8% were female, and the mean age was 41.8–42.8 years (9).

### Individual UC-related symptom resolution

**Figure 1.**
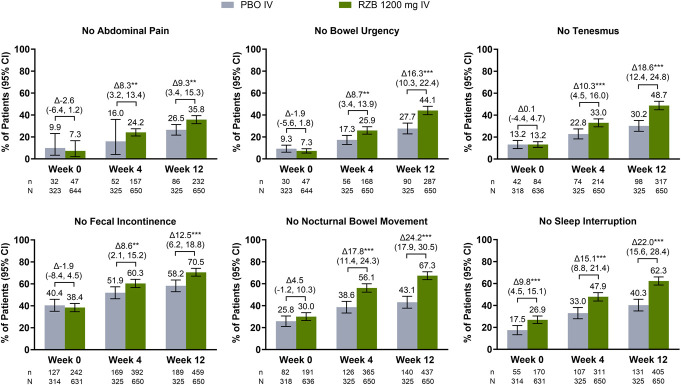
Achievement of individual symptom resolution at week 4 and week 12 of induction. CI, confidence interval; IV, intravenous; PBO, placebo; RZB, risankizumab. n = total number of patients who achieved symptom resolution; N = total number of patients assessed. **P ≤ 0.01, ***P ≤ 0.001. All *P* values in Figure 1 are considered nominal, except the *P* values for no abdominal pain, no bowel urgency, no tenesmus, and no nocturnal bowel movements at week 12, which were ranked secondary end points in INSPIRE and were considered statistically significant by the graphical multiple testing procedure.

**Figure 2.**
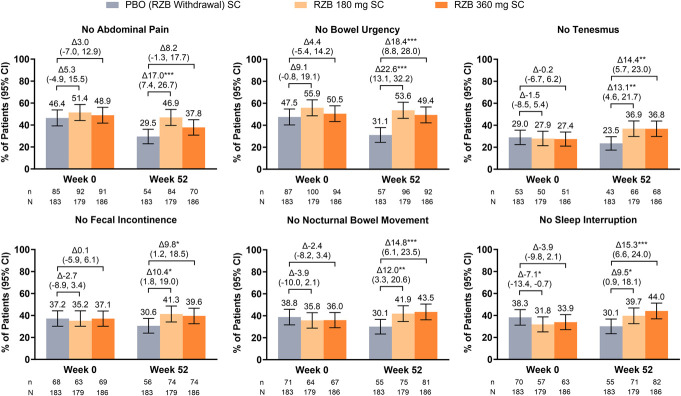
Achievement of individual symptom resolution at week 52 of maintenance. CI, confidence interval; PBO, placebo; RZB, risankizumab; SC, subcutaneous. n = total number of patients who achieved symptom resolution; N = total number of patients assessed. *P ≤ 0.05, **P ≤ 0.01, ***P ≤ 0.001. All *P* values in Figure 2 are considered nominal, except the *P* values for no abdominal pain, no bowel urgency, no tenesmus, and no nocturnal bowel movements at week 52, which were ranked secondary end points and were considered statistically significant via the graphical multiple testing procedure.

As early as week 4 of induction, greater proportions of patients treated with RZB 1,200 mg compared with patients treated with PBO experienced no abdominal pain (24.2% vs 16.0%; *P* ≤ 0.01), no bowel urgency (25.9% vs 17.3%; *P* ≤ 0.01), no tenesmus (33.0% vs 22.8%; *P* ≤ 0.001), no fecal incontinence (60.3% vs 51.9%; *P* ≤ 0.01), no nocturnal bowel movements (56.1% vs 38.6%; *P* ≤ 0.001), and no sleep interruption (47.9% vs 33.0%; *P* ≤ 0.001) (Figure 1). These benefits persisted through the end of induction with the greatest differences observed in patients who experienced no nocturnal bowel movements (67.3% vs 43.1%; *P* ≤ 0.001) and no sleep interruption (62.3% vs 40.3%; *P* ≤ 0.001) (9). Improvements in individual UC-related symptoms were observed across RZB 180 mg and RZB 360 mg at week 52 (Figure 2) (9).

### Comprehensive UC-related symptom resolution

**Figure 3.**
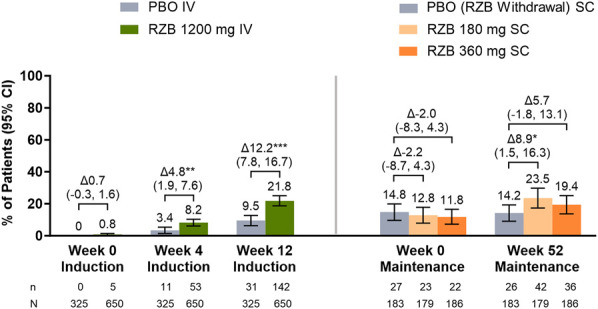
Achievement of comprehensive symptom resolution^†^ at week 4 and week 12 of induction and week 52 of maintenance. AP, abdominal pain; BU, bowel urgency; CI, confidence interval; FI, fecal incontinence; IV, intravenous; NBM, nocturnal bowel movements; PBO, placebo; RZB, risankizumab; SC, subcutaneous; SI, sleep interruption; TN, tenesmus. n = total number of patients who achieved symptom resolution; N = total number of patients assessed. ^†^Comprehensive symptom resolution is defined as no AP, no BU, no TN, no FI, no NBM, and no SI. Nominal *P ≤ 0.05, **P ≤ 0.01, ***P ≤ 0.001.

Greater proportions of patients treated with RZB achieved comprehensive symptom resolution at week 4 (8.2% vs 3.4%; *P* ≤ 0.01) and week 12 of induction (21.8% vs 9.5%; *P* ≤ 0.001) and week 52 of maintenance (RZB 180 mg, 23.5%; RZB 360 mg, 19.4%; PBO [RZB withdrawal], 14.2%; *P* ≤ 0.05 or *P* = 0.1358 vs PBO [RZB withdrawal], respectively; Figure 3).

### MWPC for HRQoL outcomes

Greater proportions of patients treated with RZB achieved MWPCs across all HRQoL outcomes at week 12 of induction (Table 1). The greatest differences were observed with RZB 1,200 mg treatment compared with PBO for patients who experienced improvements in UCSQ (63.3% vs 39.7%; *P* ≤ 0.001) and IBDQ (69.5% vs 49.2%; *P* ≤ 0.001). At week 52 of maintenance, a greater proportion of patients treated with RZB 180 mg achieved MWPCs across all HRQoL outcomes (all *P* ≤ 0.001 vs PBO [RZB withdrawal], except for overall work impairment [*P* ≤ 0.01], work time missed [*P* = 0.3324], impairment while working [*P* ≤ 0.05]; (Table 2). For RZB 360 mg, a greater proportion of patients achieved MWPCs for UCSQ, IBDQ, and SF-36 Physical Component Summary (all *P* ≤ 0.05 vs PBO [RZB withdrawal]; Table 2).

**Table 1.**
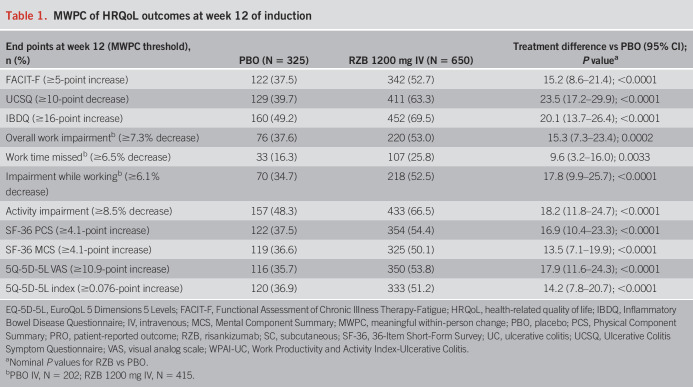
MWPC of HRQoL outcomes at week 12 of induction

**Table 2.**
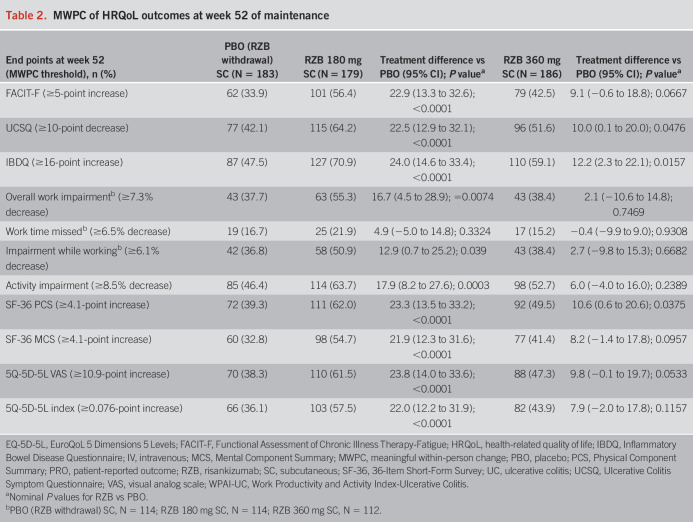
MWPC of HRQoL outcomes at week 52 of maintenance

### Sensitivity analysis for UC-related symptom resolution

As early as week 4 of induction, greater proportions of patients treated with RZB who had presented symptoms at induction baseline experienced no abdominal pain (19.8% vs 10.7%; *P* ≤ 0.001), no bowel urgency (22.5% vs 12.7%; *P* ≤ 0.001), no tenesmus (26.8% vs 16.3%; *P* ≤ 0.001), no fecal incontinence (43.8% vs 36.9%; *P* = 0.0736), no nocturnal bowel movements (42.0% vs 29.7%; *P* ≤ 0.001), and no sleep interruption (35.4% vs 27.4%; *P* ≤ 0.05; see Supplementary Figure S1, Supplementary Digital Content 1, http://links.lww.com/AJG/D606). These benefits persisted through the end of induction. Among induction responders in maintenance, improvements were greater with RZB 180 mg and 360 mg vs PBO (RZB withdrawal) for all individual symptoms at week 52 (see Supplementary Figure S2, Supplementary Digital Content 1, http://links.lww.com/AJG/D606).

### Subgroup analysis

At induction week 12, higher proportions of patients treated with RZB 1200 mg experienced improvements in all individual UC symptoms in non-AT-IR (all *P* ≤ 0.001 vs PBO, except for fecal incontinence [*P* ≤ 0.01]) and AT-IR subgroups (*P* ≤ 0.001 vs PBO; abdominal pain [*P* = 0.2716]; bowel urgency and tenesmus [*P* ≤ 0.05]; fecal incontinence [*P* ≤ 0.01]; (see Supplementary Figure S3, Supplementary Digital Content 1, http://links.lww.com/AJG/D606). After 52 weeks of maintenance treatment, more patients treated with RZB compared with patients in PBO [RZB withdrawal] reported symptom resolution in the non-AT-IR and AT-IR subgroups (see Supplementary Figure S4, Supplementary Digital Content 1, http://links.lww.com/AJG/D606). Generally, improvements were greater for UC-related symptoms in the non-AT-IR subgroup relative to the AT-IR subgroup after induction and maintenance treatment with RZB.

More patients treated with RZB achieved MWPCs relative to PBO across all HRQoL outcomes at week 12 of induction in non-AT-IR and AT-IR subgroups (see Supplementary Table S1, Supplementary Digital Content 1, http://links.lww.com/AJG/D606). At week 52 of maintenance, a greater proportion of AT-IR patients treated with RZB 180 mg or 360 mg achieved MWPCs relative to PBO across all HRQoL outcomes (see Supplementary Table S2, Supplementary Digital Content 1, http://links.lww.com/AJG/D606). For the non-AT-IR subgroup, a greater proportion of patients treated with RZB 180 mg achieved MWPCs relative to PBO across all HRQoL outcomes (see Supplementary Table S2, Supplementary Digital Content 1, http://links.lww.com/AJG/D606). A greater proportion of non-AT-IR patients treated with RZB 360 mg achieved MWPCs relative to PBO for all HRQoL outcomes, except overall work impairment, work time missed, impairment while working, and SF-36 Mental Component Summary (see Supplementary Table S2, Supplementary Digital Content 1, http://links.lww.com/AJG/D606).

## DISCUSSION

The INSPIRE and COMMAND phase 3 clinical trials assessed RZB for the treatment of patients with moderately to severely active UC and have demonstrated improved clinical remission rates at week 12 of induction and week 52 of maintenance compared with PBO (9). As multidisciplinary workflows are needed to assess treatment efficacy beyond disease activity, the current *post hoc* analysis is the first to assess the impact of RZB on PROs over time, regarding comprehensive symptom resolution, as well as HRQoL outcomes in patients with UC. Patients treated with RZB demonstrated improvements across UC-related symptoms and MWPCs for HRQoL outcomes at week 12 of induction and week 52 of maintenance compared with PBO. Improvements in individual and comprehensive UC-related symptoms were observed as early as week 4 of induction treatment. Similar findings were observed for patients in the sensitivity analysis who presented with these symptoms at baseline. MWPCs for all HRQoL outcomes were achieved at week 12 of induction and at week 52 of maintenance, with the exception of work time missed with RZB 360 mg. The AT-IR and non-AT-IR subgroups experienced greater improvements in UC-related symptoms and HRQoL with RZB treatment relative to PBO.

Previous publications have reported relationships between UC-related symptoms, such as abdominal pain and bowel urgency; social activities; mental well-being; and QoL (22,23). Bothersome symptoms of UC, such as bowel urgency, are strongly associated with active IBD and can be improved with effective treatment (1). Treatments that can reduce multiple symptoms, for example, pain and poor sleep quality, may also decrease the impact of disease on QoL and, consequently, increase the ability of patients with UC to perform work and daily activities. Ghosh et al (3) reported that patients with abdominal pain and bowel urgency at baseline had lower HRQoL scores than patients without those symptoms. They also demonstrated that patients with UC who received biologic treatment experienced improvements in abdominal pain and bowel urgency as early as week 2 compared with those who received PBO, resulting in the reduction of disease burden (3). The findings of this study demonstrate that treatment with RZB can rapidly improve symptoms, such as abdominal pain and bowel urgency, providing patients with an important option to relieve UC-related symptoms.

In this study, we sought to address a significant research gap by assessing additional novel outcome measures of tenesmus, fecal incontinence, nocturnal bowel movements, and sleep interruption that are underreported in clinical trials. Despite being underreported, these symptoms significantly affect patients' QoL (24). The findings of this study revealed that treatment with RZB not only resulted in improvements across well-established symptoms but also demonstrated positive effects on these additional novel outcome measures. Assessment of underreported symptoms of UC can provide insight into patients' perspectives of treatment efficacy, highlighting the need to prioritize research of these symptoms to ensure effective management of UC (25). The aim of the treat-to-target approach was to meaningfully adapt to the disease course of UC, restore QoL, and prevent major long-term functional impairment and disability (26). Measuring and monitoring symptoms of abdominal pain, bowel urgency, tenesmus, fecal incontinence, nocturnal bowel movements, and sleep interruption are important to help patients achieve their treatment goals of reducing UC-related symptoms (8). The findings of this study demonstrate the potential of RZB to help patients achieve STRIDE-II short-term and long-term treatment goals, for example, restoration of QoL and absence of disability (8). In addition, STRIDE-II reported that patients rated symptomatic relief as the highest immediate treatment goal (8), emphasizing the importance of monitoring and addressing burdensome symptoms. Further research is needed to examine the prolonged impact of treatment on PROs, and whether QoL continues to improve with longer treatment exposure. Additional research will deepen our understanding of the correlation between endoscopic and histologic findings and their impact on PROs.

The strengths of this study include the individual and comprehensive measurement of debilitating PROs, including the assessment of the additional novel outcome measures of tenesmus, fecal incontinence, nocturnal bowel movements, and sleep interruption, providing valuable insights into the impact of RZB induction and maintenance treatment on patients' daily lives. The outcome measures of absence of bowel urgency, absence of abdominal pain, absence of nocturnal bowel movements, absence of tenesmus, fecal incontinence, and sleep interruption were added to the trial to align with the update from STRIDE-II (8). Moreover, despite the stringency, approximately 20% of patients in induction and maintenance, respectively, achieved comprehensive symptom resolution of all 6 outcome measures. This indicates that RZB treatment improved multiple symptoms effectively, which is a positive outcome within the context of these stringent parameters. This information can be invaluable for healthcare providers in making informed treatment decisions and improving patient care. By examining the QoL of patients undergoing RZB treatment, this study sheds light on the broader implications and benefits of RZB beyond objective clinical measures. Limitations of the study include the sample size and that the assessments include *post hoc* analyses. Data in the real-world setting and beyond 52 weeks of treatment are needed to assess the benefit of RZB therapy on symptom resolution and improvements in HRQoL in patients with UC. Moreover, further research is critical to better understand the correlation between these symptoms and clinical outcomes and to determine which improvements in clinical outcomes have the most impact on patient symptoms and overall QoL.

Patients treated with RZB experienced improvements in individual and comprehensive UC-related symptoms, as well as MWPCs of HRQoL outcomes, when compared with PBO during induction and maintenance. Improvements in symptoms were observed as early as week 4 of induction treatment with RZB vs PBO and continued through 52 weeks of maintenance treatment. Therefore, the results of this study support the use of RZB as a treatment option to improve burdensome symptoms and achieve meaningful improvements in QoL for patients with moderately to severely active UC.

## CONFLICTS OF INTEREST

**Guarantor of the article:** Remo Panaccione, MD.

**Specific author contributions:** J.T., J.P., C.A.S., M.C.D., P.S.D., S.T., S.D., and R.P. participated equally in the data acquisition. J.T., J.P., C.A.S., M.C.D., P.S.D., S.T., S.D., J.K., J.L., N.S., L.R., C.H., D.S., and R.P. participated equally in the study design. J.T., J.K., J.L., N.S., L.R., C.H., D.S., and R.P. participated equally in the assessment and verification of the data. All authors had access to relevant data, participated in data interpretation, critically reviewed the manuscript, and provided final approval for publication. No honoraria or payments were made for authorship.

**Financial support:** AbbVie funded the INSPIRE (NCT03398148) and COMMAND (NCT03398135) studies and participated in the study design, research, analysis, data collection, interpretation of data, reviewing, and approval of this publication. All authors had access to relevant data and participated in the drafting, review, and approval of this publication. No honoraria or payments were made for authorship. AbbVie and the authors thank all trial investigators and the patients who participated in these clinical trials. Medical writing support was provided by Doreen Kruk of Avalere Health, and funded by AbbVie, and Kristina Y. Aguilera, PhD, of AbbVie. Editorial support was provided by Angela T. Hadsell of AbbVie.

**Potential competing interests:** J.T. has received consulting fees from Janssen, AbbVie, Lilly, Sandoz, and Pfizer and has received research funding from AbbVie and Janssen. J.P. has received consultancy fees/honorarium from AbbVie, Alimentiv, Athos, Atomwise, Boehringer Ingelheim, Celsius, Ferring, Galapagos, Genentech/Roche, GlaxoSmithKline, Janssen, Mirum, Nimbus, Pfizer, Progenity, Prometheus, Protagonist, Revolo, Sanofi, Sorriso, Surrozen, Takeda, and Wasserman, and has served on data safety monitoring boards for Alimentiv, Mirum, Roche, Sorriso, Sanofi, and Surrozen. C.A.S. has served as a consultant to AbbVie, BMS, Boomerang, Buhlmann, Lilly, Johnson & Johnson, Napo Pharmaceuticals, Pfizer, Prometheus Biosciences, Takeda, and Trellus Health, as a speaker for AbbVie, Janssen, Pfizer, and Takeda, and received grant support from AbbVie, Janssen, Lilly, Pfizer, and Takeda. M.C.D. has served as a consultant and advisor for AbbVie, Abivax, Astra Zeneca, Arena, BMS, Celltrion, Eli Lilly Gilead, Genentech, Janssen, Johnson and Johnson, Merck, Pfizer, Prometheus Labs, Sanofi, Spyre and Takeda. P.S.D. has served on advisory boards for and/or has received research support and/or speaker/consultation fees from AbbVie, Abivax, Adiso, Bristol Myers Squibb, Geneoscopy, GlaxoSmithKline, Janssen, Eli Lilly, Pfizer, Roivant, and Takeda. S.T. has received speaker fees from JIMRO, Mitsubishi Tanabe Pharma, AbbVie, Janssen, Kissei Pharmaceutical, and writing fees from Pfizer. S.D. has served a consultant for AbbVie, Allergan, Amgen, AstraZeneca, Biogen, Boehringer Ingelheim, Celgene, Celltrion, Eli Lilly, Enthera, Ferring, Gilead, Hospira, Janssen, Johnson & Johnson, Merck, Mundipharma, Mylan, Pfizer, Roche, Sandoz, Sublimity, Takeda, TiGenix, UCB, and Vifor. J.-H.L. has no conflict of interest to declare. J.K., N.S., L.R., C.H., and D.S. are employees of AbbVie and may own stock options. R.P. has consulted for Abbott, AbbVie, Abbivax, Alimentiv (formerly Robarts), Amgen, AnaptysBio, Arena Pharmaceuticals, AstraZeneca, Biogen, Boehringer Ingelheim, Bristol-Myers Squibb, Celgene, Celltrion, Cosmos Pharmaceuticals, Eisai, Elan, Eli Lilly, Ferring, Galapagos, Fresenius Kabi, Genentech, Gilead Sciences, Glaxo-Smith Kline, JAMP Bio, Janssen, Merck, Mylan, Novartis, Oppilan Pharma, Organon, Pandion Pharma, Pendopharm, Pfizer, Progenity, Prometheus Biosciences, Protagonist Therapeutics, Roche, Sandoz, Satisfai Health, Shire, Sublimity Therapeutics, Spyre Therapeutics, Takeda Pharmaceuticals, Theravance Biopharma, Trellus, Union Biopharma, Viatris, Ventyx, UCB and served as a speaker for AbbVie, Amgen, Arena Pharmaceuticals, Bristol-Myers Squibb, Celgene, Eli Lilly, Ferring, Fresenius Kabi, Gilead Sciences, Janssen, Merck, Organon, Pfizer, Roche, Sandoz, Shire, Takeda Pharmaceuticals and participated in advisory boards for AbbVie, Alimentiv (formerly Robarts), Amgen, Arena Pharmaceuticals, AstraZeneca, Biogen, Boehringer Ingelheim, Bristol-Myers Squibb, Celgene, Eli Lilly, Ferring, Fresenius Kabi, Genentech, Gilead Sciences, Glaxo-Smith Kline, JAMP Bio, Janssen, Merck, Mylan, Novartis, Oppilan Pharma, Organon, Pandion Pharma, Pfizer, Progenity, Protagonist Therapeutics, Roche, Sandoz, Shire, Sublimity Therapeutics, Takeda Pharmaceuticals, Ventyx.

**Clinical trials:** NCT03398148; NCT03398135.

**Study Highlights**

**WHAT IS KNOWN**

✓ Patients with ulcerative colitis (UC) experience disruptive symptoms that can impair their quality of life.
✓ Risankizumab improves clinical, endoscopic, and histologic outcomes for patients with UC.

**WHAT IS NEW HERE**

✓ This analysis assesses risankizumab for UC on patient-reported outcomes during induction and maintenance treatment.
✓ This study includes the evaluation of novel patient-reported outcome measures (i.e., tenesmus, fecal incontinence, nocturnal bowel movements, and sleep interruption) and comprehensive symptom resolution.
✓ In patients with moderately to severely active UC, risankizumab treatment provides comprehensive improvement in both symptoms and meaningful health-related quality-of-life outcomes.

## Supplementary Material
