# Supplementary material for: Symptom Resolution and Meaningful Improvement in Quality of Life With Risankizumab in Patients With Ulcerative Colitis: Post Hoc Analysis of the Randomized INSPIRE and COMMAND Studies
Source: Am J Gastroenterol. 2025 Mar 17;120(8):1820–8. doi: 10.14309/ajg.0000000000003420 (PMC12282589; doi:10.14309/ajg.0000000000003420)
Supplement: Supplementary file 1 [file acg-120-1820-s001.docx]

**Figure S1.** **Achievement of Individual Symptom Resolution at Week 4 and Week 12 of Induction in Patients With Symptoms at Baseline**


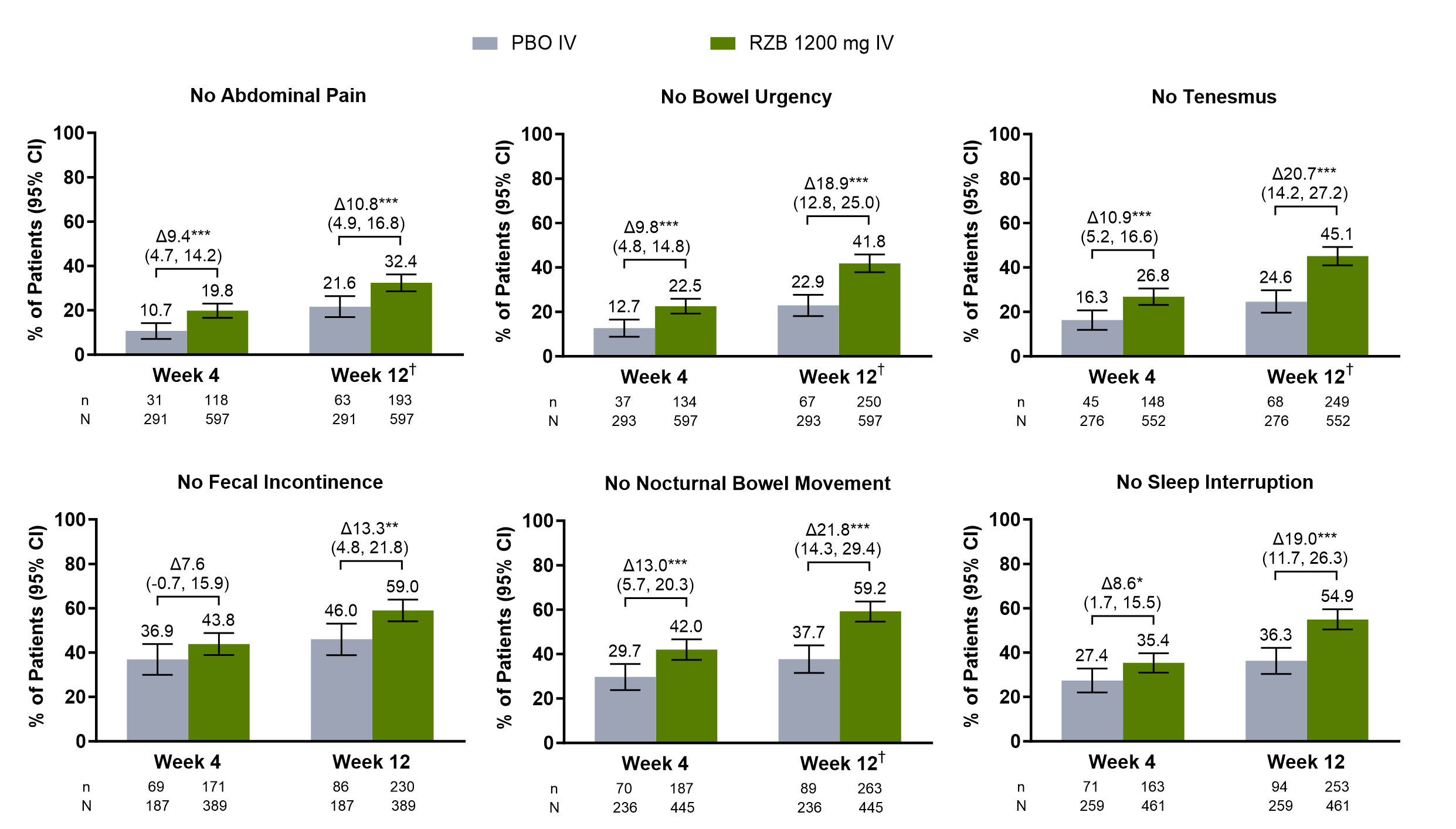


CI, confidence interval; IV, intravenous; PBO, placebo; RZB, risankizumab. n = total number of patients who achieved symptom resolution; N = total number of patients assessed. Nominal **P* ≤ .05, ***P* ≤ .01, ****P* ≤ .001. ^†^Adapted from Louis E, et al. JAMA. 2024;332(11):881–897.

**Figure S2.** **Achievement of Individual Symptom Resolution at Week 52 of Maintenance in Patients With Symptoms at Baseline**


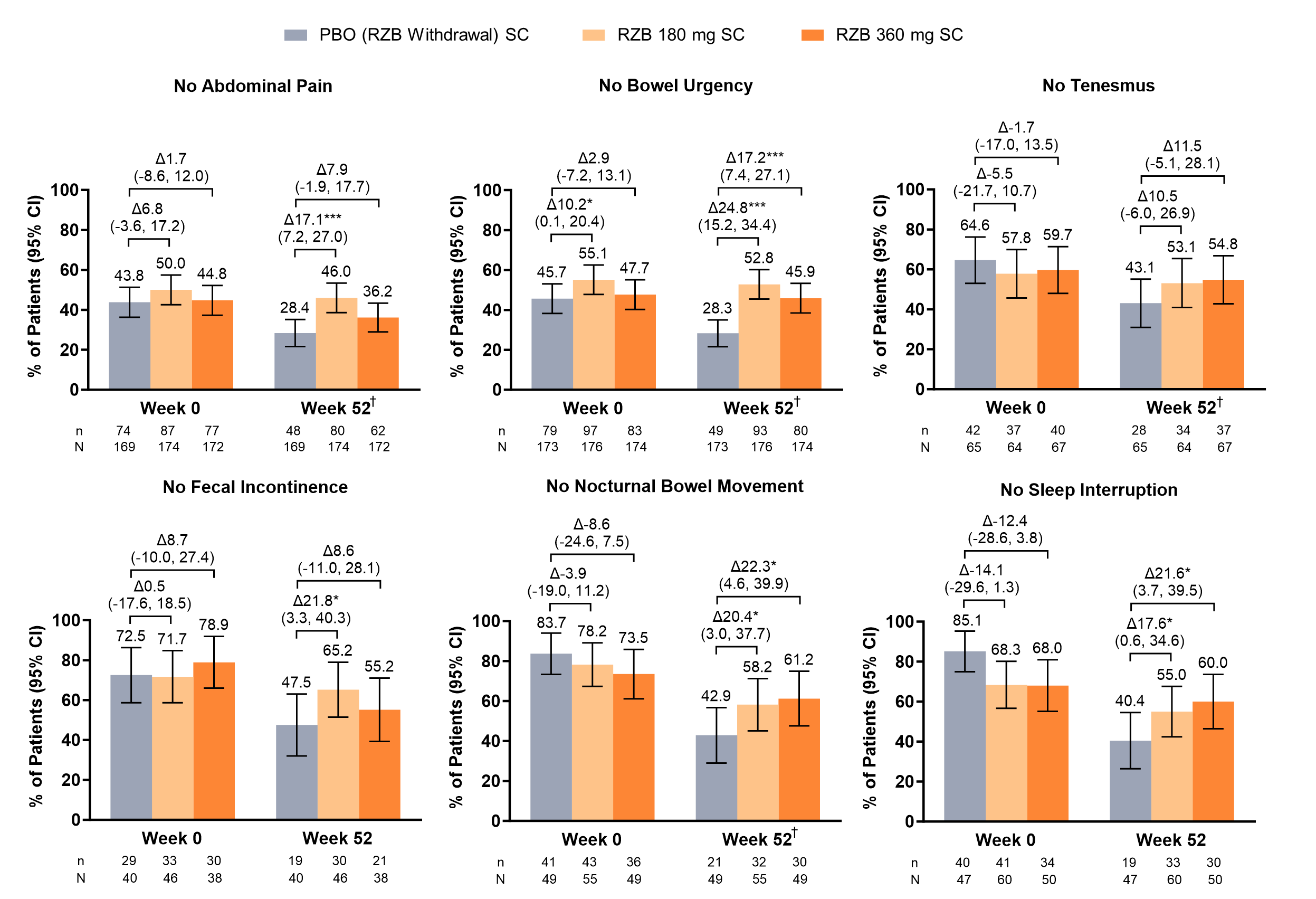


CI, confidence interval; PBO, placebo; RZB, risankizumab; SC, subcutaneous. n = total number of patients who achieved symptom resolution; N = total number of patients assessed. Nominal **P* ≤ .05, *** *P* ≤ .001. ^†^Adapted from Louis E, et al. JAMA. 2024;332(11):881–897.

**Figure S3. Achievement of Individual Symptom Resolution at Week 12 of Induction by Prior Exposure to Advanced Therapy**


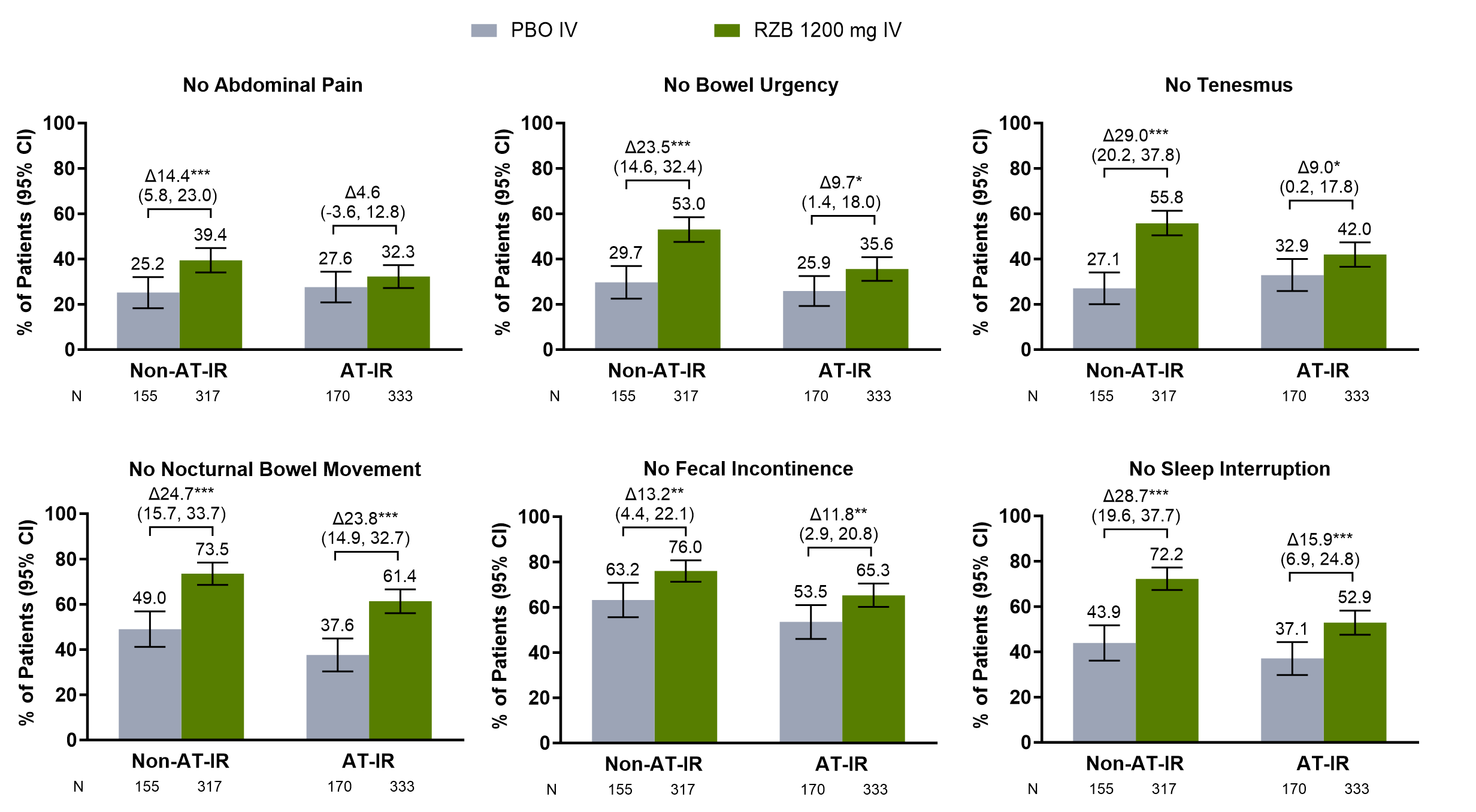


AT, advanced therapy; IR, intolerance or inadequate response; IV, intravenous; PBO, placebo; RZB, risankizumab. N = total number of patients assessed. Nominal **P* < .05; ***P* < .01; *** *P* < .001.

**Figure S4: Achievement of Individual Symptom Resolution at Week 52 of Maintenance by Prior Exposure to Advanced Therapy**


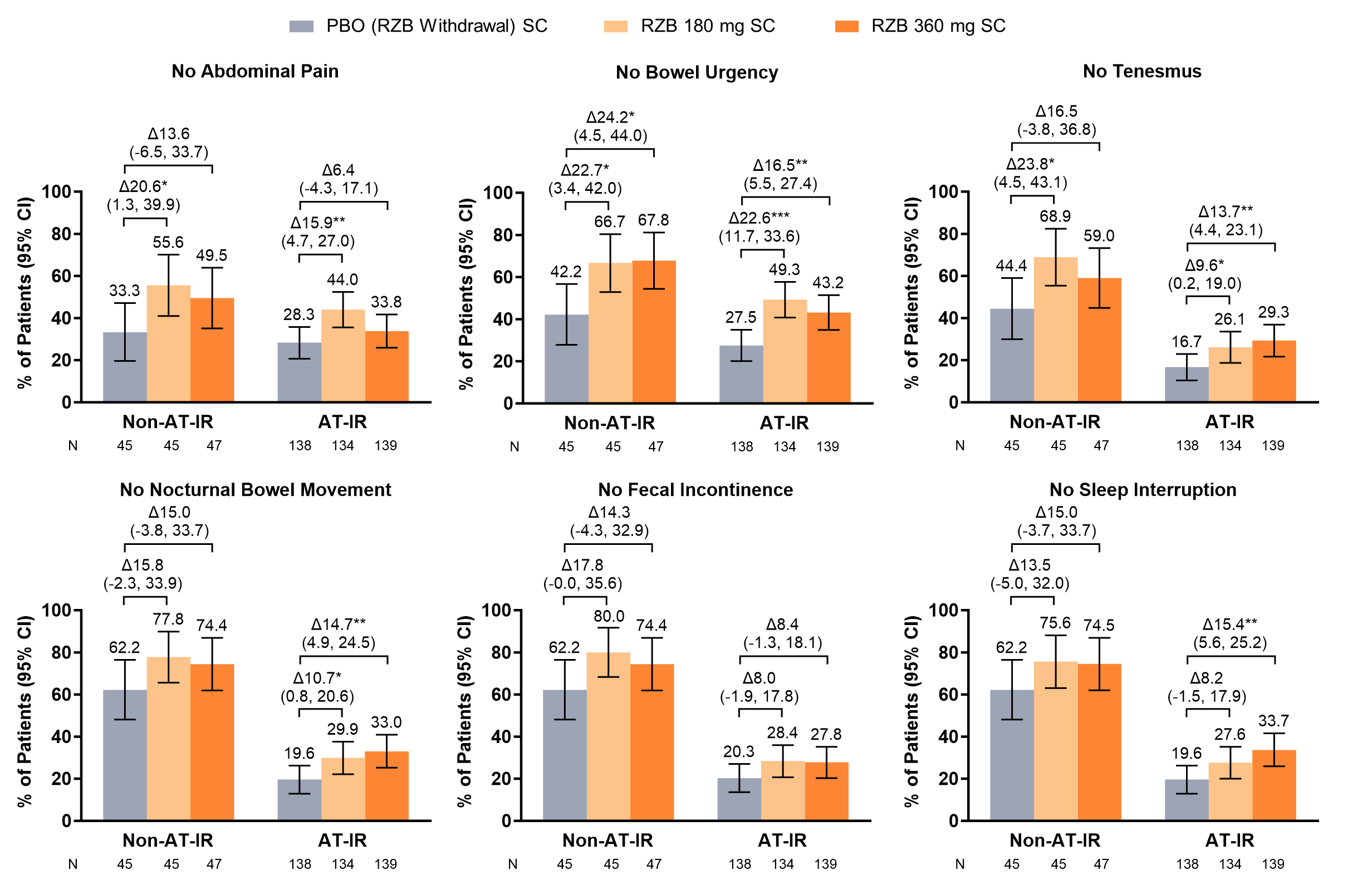


AT, advanced therapy; IR, intolerance or inadequate response; PBO, placebo; RZB, risankizumab; SC, subcutaneous. N = total number of patients assessed. Nominal **P* < .05; ***P* < .01; ****P* < .001

**Table S1. MWPC of HRQoL Outcomes at Week 12 of Induction by Prior Exposure to Advanced Therapy**

|  | **Non-AT-IR** | | | **AT-IR** | | |
| --- | --- | --- | --- | --- | --- | --- |
| **Endpoints at Week 12 (MWPC Threshold), n (%)** | **PBO**  **N = 155** | **RZB 1200 mg IV**  **N = 317** | **Treatment Difference vs PBO (95% CI); *P* Value**^†^ | **PBO**  **N = 170** | **RZB 1200 mg IV**  **N = 333** | **Treatment Difference vs PBO (95% CI); *P* Value**^†^ |
| FACIT-F (≥ 5-point increase) | 66 (42.6) | 172 (54.3) | 11.5 (2.1, 20.8);  *P* = .0161 | 56 (32.9) | 170 (51.2) | 18.2 (9.4, 27.0);  *P* < .0001 |
| UCSQ (≥ 10-point decrease) | 65 (41.9) | 223 (70.3) | 28.4 (19.3, 37.5);  *P* < .0001 | 64 (37.6) | 188 (56.6) | 19.0 (10.1, 28.0);  *P* < .0001 |
| IBDQ (≥ 16-point increase) | 83 (53.5) | 245 (77.3) | 23.6 (14.8, 32.4);  *P* < .0001 | 77 (45.3) | 207 (62.0) | 16.8 (7.7, 25.9);  *P* = .0003 |
| Overall work impairment^‡^ (≥ 7.3% decrease) | 40 (39.2) | 117 (55.7) | 16.2 (4.9, 27.6);  *P* = .0050 | 36 (36.0) | 103 (50.1) | 14.5 (3.1, 25.8);  *P* = .0129 |
| Work time missed^‡^ (≥ 6.5% decrease) | 19 (18.6) | 55 (26.2) | 7.8 (-1.4, 16.9);  *P* = .0953 | 14 (14.0) | 52 (25.4) | 11.4 (2.5, 20.4);  *P* = .0122 |
| Impairment while working^‡^ (≥ 6.1% decrease) | 38 (37.3) | 116 (55.2) | 17.5 (6.4, 28.7);  *P* = .0021 | 32 (32.0) | 102 (49.7) | 18.0 (6.8, 29.2);  *P* = .0016 |
| Activity impairment (≥ 8.5% decrease) | 79 (51.0) | 219 (69.1) | 18.2 (9.0, 27.4);  *P* = .0001 | 78 (45.9) | 214 (64.1) | 18.2 (9.2, 27.3);  *P* < .0001 |
| SF-36 PCS (≥ 4.1-point increase) | 64 (41.3) | 184 (58.0) | 16.8 (7.4, 26.2);  *P* = .0005 | 58 (34.1) | 170 (51.0) | 16.9 (8.0, 25.8);  *P* = .0002 |
| SF-36 MCS (≥ 4.1-point increase) | 62 (40.0) | 173 (54.6) | 14.8 (5.4, 24.1);  *P* = .0019 | 57 (33.5) | 153 (45.8) | 12.3 (3.4, 21.1);  *P* = .0066 |
| 5Q-5D-5L VAS (≥ 10.9-point increase) | 62 (40.0) | 185 (58.4) | 18.2 (8.9, 27.5);  *P* = .0001 | 54 (31.8) | 165 (49.4) | 17.7 (9.0, 26.5);  *P* < .0001 |
| 5Q-5D-5L Index (≥ 0.076-point increase) | 60 (38.7) | 174 (54.9) | 16.2 (6.8, 25.5);  *P* = .0007 | 60 (35.3) | 159 (47.7) | 12.4 (3.5, 21.3);  *P* = .0062 |

AT, advanced therapy; EQ-5D-5L, EuroQoL 5 Dimensions 5 Levels; FACIT-F, Functional Assessment of Chronic Illness Therapy-Fatigue; HRQoL, health-related quality of life; IBDQ, Inflammatory Bowel Disease Questionnaire; IR, intolerance or inadequate response; IV, intravenous; MCS, Mental Component Summary; MWPC, meaningful within-person change; PBO, placebo; PCS, physical component summary; PRO, patient-reported outcome; RZB, risankizumab; SC, subcutaneous; SF-36, 36-Item Short-Form Survey; UC, ulcerative colitis; UCSQ, Ulcerative Colitis Symptom Questionnaire; VAS, visual analog scale; WPAI-UC, Work Productivity and Activity Index-Ulcerative Colitis. ^†^Nominal *P* values for RZB versus PBO. ^‡^Non-AT-IR: PBO IV, N = 102; RZB 1200 mg IV, N = 210; AT-IR: PBO IV, N = 100; RZB 1200 mg IV, N = 205.

**Table S2. MWPC of HRQoL Outcomes at Week 52 of Maintenance by Prior Exposure to Advanced Therapy**

|  | **Non-AT-IR** | | | | | **AT-IR** | | | | |
| --- | --- | --- | --- | --- | --- | --- | --- | --- | --- | --- |
| **Endpoints at Week 52 (MWPC Threshold), n (%)** | **PBO**  **(RZB Withdrawal) SC**  **N = 45** | **RZB 180 mg SC**  **N = 45** | **Treatment Difference vs PBO (95% CI); *P* Value**^†^ | **RZB 360 mg SC**  **N = 47** | **Treatment Difference vs PBO (95% CI); *P* Value**^†^ | **PBO**  **(RZB Withdrawal) SC**  **N = 138** | **RZB 180 mg SC**  **N = 134** | **Treatment Difference vs PBO (95% CI); *P* Value**^†^ | **RZB 360 mg SC**  **N = 139** | **Treatment Difference vs PBO (95% CI); *P* Value**^†^ |
| FACIT-F  (≥ 5-point increase) | 17 (37.8) | 30 (66.7) | 28.5 (8.8, 48.2);  *P* = .0045 | 21 (44.5) | 6.6 (-13.3, 26.4);  *P* = .5177 | 45 (32.6) | 71 (53.0) | 21.1 (10.0, 32.2);  *P* = .0002 | 58 (41.7) | 9.9 (-1.3, 21.0);  *P* = .0824 |
| UCSQ (≥ 10-point decrease) | 26 (57.8) | 36 (80.0) | 20.6 (2.5, 38.7);  *P* = .0261 | 28 (59.5) | 0.2 (-19.6, 20.1);  *P* = .9829 | 51 (37.0) | 79 (59.0) | 23.2 (11.9, 34.5);  *P* < .0001 | 68 (48.9) | 13.2 (1.8, 24.7);  *P* = .0236 |
| IBDQ (≥ 16-point increase) | 29 (64.4) | 40 (88.9) | 24.0 (7.3, 40.7);  *P* = .0048 | 32 (68.1) | 3.1 (-16.1, 22.4);  *P* = .7506 | 58 (42.0) | 87 (64.9) | 24.0 (12.7, 35.3);  *P* < .0001 | 78 (56.1) | 15.2 (3.6, 26.7);  *P* = .0100 |
| Overall work impairment^‡^  (≥ 7.3% decrease) | 13 (48.1) | 28 (77.8) | 30.3 (7.8, 52.8);  *P* = .0082 | 11 (39.3) | -7.3 (-34.3, 19.7);  *P* = .5947 | 30 (34.5) | 35 (44.9) | 11.4 (-3.1, 26.0);  *P* = .1231 | 32 (38.1) | 5.0 (-9.4, 19.3);  *P* = .4952 |
| Work time missed^‡^ (≥ 6.5% decrease) | 6 (22.2) | 9 (25.0) | 1.1 (-19.8, 22.0);  *P* = .9180 | 3 (10.7) | -10.8 (-31.8, 10.2);  *P* = .3146 | 13 (14.9) | 16 (20.5) | 6.4 (-4.7, 17.4);  *P* = .2605 | 14 (16.7) | 2.8 (-7.8, 13.3);  *P* = .6080 |
| Impairment while working^‡^ (≥ 6.1% decrease) | 14 (51.9) | 25 (69.4) | 18.3 (-5.2, 41.8);  *P* = .1270 | 13 (46.4) | -3.7 (-31.0, 23.5);  *P* = .7886 | 28 (32.2) | 33 (42.3) | 10.8 (-3.5, 25.2);  *P* = .1396 | 30 (35.7) | 4.7 (-9.4, 18.9);  *P* = .5105 |
| Activity impairment  (≥ 8.5% decrease) | 27 (60.0) | 35 (77.8) | 18.9 (1.1, 36.7);  *P* = .0370 | 29 (61.7) | 0.4 (-19.2, 20.0);  *P* = .9706 | 58 (42.0) | 79 (59.0) | 17.5 (6.1, 29.0);  *P* = .0027 | 69 (49.6) | 7.9 (-3.8, 19.5);  *P* = .1857 |
| SF-36 PCS  (≥ 4.1-point increase) | 22 (48.9) | 32 (71.1) | 23.6 (4.1, 43.1);  *P* = .0179 | 27 (57.4) | 10.4 (-10.0, 30.7);  *P* = .3180 | 50 (36.2) | 79 (59.0) | 23.3 (11.9, 34.7);  *P* < .0001 | 65 (46.8) | 10.7 (-0.8, 22.1);  *P* = .0679 |
| SF-36 MCS  (≥ 4.1-point increase) | 22 (48.9) | 30 (66.7) | 17.4 (-2.4, 37.1);  *P* = .0843 | 23 (48.9) | -0.7 (-21.0, 19.6);  *P* = .9482 | 38 (27.5) | 68 (50.7) | 23.4 (12.3, 34.5);  *P* < .0001 | 54 (38.8) | 11.1 (0.2, 22.0);  *P* = .0467 |
| 5Q-5D-5L VAS  (≥ 10.9-point increase) | 22 (48.9) | 34 (75.6) | 26.3 (7.0, 45.6);  *P* = .0076 | 24 (51.1) | 2.6 (-17.9, 23.1);  *P* = .8056 | 48 (34.8) | 76 (56.7) | 23.0 (11.7, 34.3);  *P* < .0001 | 64 (46.0) | 12.2 (0.8, 23.5);  *P* = .0360 |
| 5Q-5D-5L Index (≥ 0.076-point increase) | 21 (46.7) | 31 (68.9) | 22.6 (3.1, 42.1);  *P* = .0229 | 23 (48.3) | 1.3 (-19.3, 21.8);  *P* = .9049 | 45 (32.6) | 72 (53.7) | 21.8 (10.5, 33.2);  *P* = .0002 | 59 (42.4) | 10.1 (-1.2, 21.4);  *P* = .0785 |

AT, advanced therapy; EQ-5D-5L, EuroQoL 5 Dimensions 5 Levels; FACIT-F, Functional Assessment of Chronic Illness Therapy-Fatigue; HRQoL, health-related quality of life; IBDQ, Inflammatory Bowel Disease Questionnaire; IR, intolerance or inadequate response; IV, intravenous; MCS, Mental Component Summary; MWPC, meaningful within-person change; PBO, placebo; PCS, physical component summary; PRO, patient-reported outcome; RZB, risankizumab; SC, subcutaneous; SF-36, 36-Item Short-Form Survey; UC, ulcerative colitis; UCSQ, Ulcerative Colitis Symptom Questionnaire; VAS, visual analog scale; WPAI-UC, Work Productivity and Activity Index-Ulcerative Colitis. ^†^Nominal *P* values for RZB versus PBO. ^‡^Non-AT-IR: PBO (RZB withdrawal) SC, N = 27; RZB 180 mg SC, N = 36; RZB 360 mg SC, N = 28; AT-IR: PBO (RZB withdrawal) SC, N = 87; RZB 180 mg SC, N = 78; RZB 360 mg SC, N = 84.
